# Trichostatin A Selectively Suppresses the Cold-Induced Transcription of the *ZmDREB1* Gene in Maize

**DOI:** 10.1371/journal.pone.0022132

**Published:** 2011-07-21

**Authors:** Yong Hu, Lu Zhang, Lin Zhao, Jun Li, Shibin He, Kun Zhou, Fei Yang, Min Huang, Li Jiang, Lijia Li

**Affiliations:** State Key Laboratory of Hybrid Rice, College of Life Sciences, Wuhan University, Wuhan, People's Republic of China; Oregon State University, United States of America

## Abstract

Post-translational modifications of histone proteins play a crucial role in responding to environmental stresses. Histone deacetylases (HDACs) catalyze the removal of an acetyl group from histones and are generally believed to be a transcriptional repressor. In this paper, we report that cold treatment highly induces the up-regulation of HDACs, leading to global deacetylation of histones H3 and H4. Treatment of maize with the HDAC inhibitor trichostatin A (TSA) under cold stress conditions strongly inhibits induction of the maize cold-responsive genes *ZmDREB1* and *ZmCOR413*. However, up-regulation of the *ZmICE1* gene in response to cold stress is less affected. The expression of drought and salt induced genes, *ZmDBF1* and *rab17*, is almost unaffected by TSA treatment. Thus, these observations show that HDACs may selectively activate transcription. The time course of TSA effects on the expression of *ZmDREB1* and *ZmCOR413* genes indicates that HDACs appear to directly activate the *ZmDREB1* gene, which in turn modulates *ZmCOR413* expression. After cold treatment, histone hyperacetylation and DNA demethylation occurs in the ICE1 binding region, accompanied by an increase in accessibility to micrococcal nuclease (MNase). The two regions adjacent to the ICE1 binding site remain hypoacetylated and methylated. However, during cold acclimation, TSA treatment increases the acetylation status and accessibility of MNase and decreases DNA methylation at these two regions. However, TSA treatment does not affect histone hyperacetylation and DNA methylation levels at the ICE1 binding regions of the *ZmDREB1* gene. Altogether, our findings indicate that HDACs positively regulate the expression of the cold-induced *ZmDREB1* gene through histone modification and chromatin conformational changes and that this activation is both gene and site selective.

## Introduction

Plants regulate the expression of some genes when they encounter diverse environmental stresses, such as low temperature, drought or high salinity. Cold stress induces gene expression largely through an abscisic acid (ABA)-independent signaling pathway. In this cold responsive signaling pathway, genes encoding for transcription factors are particularly pivotal because they regulate the expression of the target genes mediating the adaptive response to cold stress. Among these genes, DREB1 (dehydration responsive element binding 1) proteins have been demonstrated to play an important role in the response of plants to cold stress [1]. In Arabidopsis, three closely related *DREB1/CBF* genes, *DREB1B/CBF1*, *DREB1A/CBF3* and *DREB1C/CBF2*, are well studied and located in a tandem repeat on chromosome 4 [2]. In maize, the *ZmDREB1* gene, characterized by high homology with the *DREB1* gene of Arabidopsis, is present as a single locus in the genome, and it is mapped on chromosome 6 [3], [4]. The DREB1 transcription factor activates the transcription of target genes, such as *COR* (cold-regulated), through binding the CRT/DRE cis-acting element in the promoter regions of these target genes [5], [6]. ICE1 (inducer of CBF/DREB expression 1) is first activated by cold stress and is an upstream transcription factor controlling the expression of the *DREB1* gene [7]. More recently, Arabidopsis studies have shown that histone acetylation/deacetylation is involved in the stress response in plants. For example, genetic analysis indicated that Arabidopsis HOS15 confers tolerance to cold through histone deacetylation [8]. Transgenic Arabidopsis plants over-expressing AtHD2C showed greater tolerance to salt and drought stresses than wild-type plants [9]. Similarly, it has been demonstrated that HDA19 may play a key role in responses to pathogens [10]. Targeted recruitment of histone acetyltransferases (HATs) to promoters often leads to localized histone acetylation at the promoter regions and is generally considered to be associated with transcriptionally active genes [11], [12]. Indeed, HATs such as GCN5 may be recruited through the CBF1 transcription factor to induce the expression of cold-regulated genes [13]. Mutations in *GCN5* and *ADA2* decrease the expression of the cold-responsive genes and, therefore, Arabidopsis cold tolerance [14]. Conversely, the histone deacetylase (HDAC) catalyzes the removal of acetyl groups from histones, and it is classically believed to repress gene expression [15], [16]. For example, the Arabidopsis ERF7 transcriptional repressor interacts with the HDA19 histone deacetylase to repress the transcription of some stress response genes [17]. However, the impact of HDACs on cold-inducible gene expression has not been intensively studied, and much less is understood regarding its functions and mechanisms in this regulation process.

Small-molecule chemical inhibitors rapidly inhibit enzyme activity in cells, which allows analysis of the time course of change and immediate assessment of the drug effects [18]. The small molecule HDAC inhibitor trichostatin A (TSA) represses histone deacetylation, resulting in histone hyperacetylation [18], [19]. Although it is generally accepted that HDACs are negative regulators of gene expression, it has been reported that HDAC inhibitors decrease gene expression in yeast, human and animal culture cells [18], [20]. In the present study, we investigate the function of HDACs during cold acclimation in maize. Our results demonstrate that TSA selectively suppresses the induction of the cold-responsive transcription factor gene *ZmDREB1* through histone modification in the defined promoter region, resulting in reduced transcription of its target gene, *ZmCOR413*.

## Results

### Histones H3 and H4 are globally deacetylated after treatment with cold (4°C)

**Figure 1:**
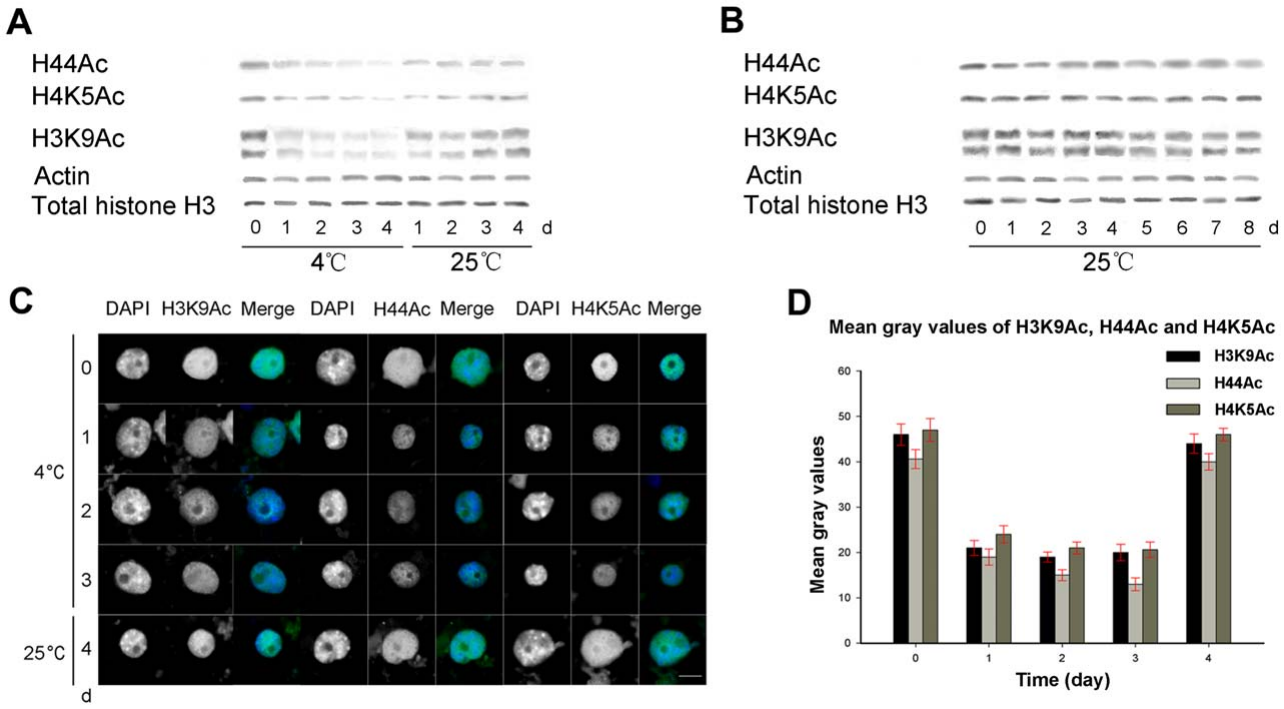
Western blot assay demonstrates the change in histone acetylation levels during cold acclimation. A. Protein samples were extracted from 7-day-old seedlings grown under 25°C (0 day), from seedlings that were further cold-treated at for 1–4 days, and from seedlings returned to 25°C for 1–4 days after cold treatment. Global acetylation levels of histones H3 and H4 were decreased after cold treatment and were recovered when seedlings were returned to 25°C. B. Reference cultivation without cold treatment was performed in parallel, showing that the histone acetylation levels underwent little change. Beta actin and total histone H3 were used as equal loading controls, and representative blots are shown. C. Deacetylation of histones H3 and H4 is revealed by immunostaining representative interphase nuclei with specific antibodies. DAPI was used as a counterstain. The ‘DAPI’ panel shows DAPI-stained DNA images, the ‘H3K9Ac’, ‘H44Ac’ and ‘H4K5Ac’ panels show immunostained images, and the ‘Merge’ panel shows a combination of blue and green signals. Under normal growth conditions, the strongly acetylated histones H3 and H4 signals were evenly distributed in the nucleus, and nucleoli were barely acetylated. When deacetylation of histones H3 and H4 occurred, weak acetylation signals were observed during cold acclimation. The histone acetylation levels were recovered after the seedlings were returned to 25°C (Bar = 10 µm). D. Histogram showing the mean gray values of the immunostaining signals for histone acetylation. Error bars represent the standard error of the mean. More than 500 nuclei were analyzed.

Recent studies have demonstrated that histone acetylation of chromatin is involved in plant responses to environmental stress [21]. To investigate dynamic changes in histone acetylation under cold stress conditions, we performed western blot detection on the levels of H3K9Ac, H4K5Ac and H44Ac in both normal and cold-treated maize seedlings prepared at various time points. As shown in Figure 1, there were significantly decreased levels of acetylated histones in cold-treated plants (Figure 1A) compared with plants grown at normal temperatures (Figure 1B), indicating that cold stress induced a global decrease in the acetylation levels of histones H3 and H4. The overall acetylation levels were rapidly recovered when cold stress was removed (Figure 1A). Plants grown under normal temperatures showed that acetylation levels were not significantly altered during growth (Figure 1B), suggesting that the deacetylation was solely due to the effects of cold stress. Further *in situ* chromatin immunostaining of interphase nuclei showed that the signals for strongly acetylated H3 and H4 histones were evenly dispersed in the nucleus at interphase, and nucleoli were barely acetylated (Figure 1C). In comparison, acetylation signal intensity was reduced after cold treatment, indicating that deacetylation of histones H3 and H4 occurred during cold acclimation (Figure 1C). Quantification of the signal intensity by measuring mean gray values showed that histone acetylation was decreased by approximately 50% after treatment with cold (Figure 1D). The chromatin reverted to the normal acetylation state when seedlings were returned to 25°C (Figure 1D). As a control, immunostaining using only a secondary antibody showed no specific labeling of nuclei (data not shown).

**Figure 2:**
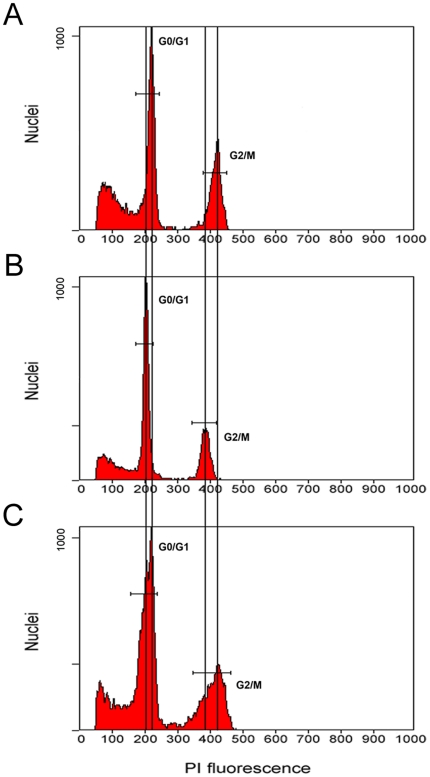
Genome-wide chromatin condensation revealed under cold stress. Nuclei were prepared from seedlings grown under normal (A) and cold stress conditions (B) and stained with PI. C. Flow cytometric analysis of a mixture of both types of nuclei. Note that the PI fluorescence peak displayed a slight leftward shift in cold-acclimated plants compared with a control group.

Chromatin condensation has been shown to be associated with histone deacetylation. Because PI (propidium iodide) fluorescence intensity is directly proportional to the level of accessible DNA and reflects the condensation state of chromatin [22], a FACS flow cytometer was used to examine chromatin conformational changes during cold acclimation. Flow cytometric analysis showed specific cold-induced changes in PI fluorescence intensity. After cold treatment, the nuclei reproducibly showed a slight reduction in fluorescence intensity and a left shift in the flow peak (Figure 2B) compared with the control (Figure 2A). Mixing analysis of control and cold-treated nuclei showed that each population still retained its position in the FACS histogram, confirming that the shift is indeed an inherent feature rather than an experimental error (Figure 2C). When cold-treated plants were returned to culturing in warm temperatures for 24 h, flow peaks returned to the control positions. These results revealed that alterations in overall chromatin conformation may occur after cold treatment, thus leading to changes in the accessibility of DNA to PI.

### HDAC expression levels are increased during cold acclimation

**Figure 3:**
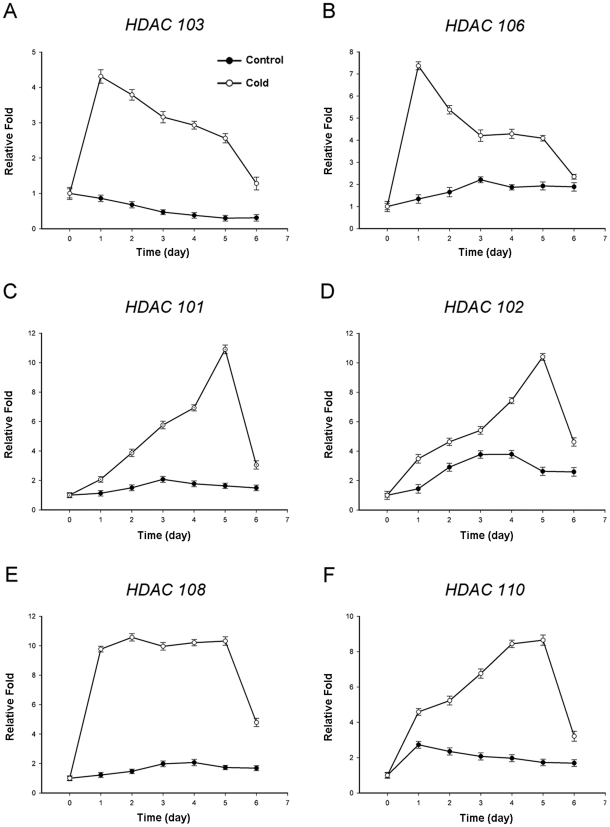
The transcriptional profile of HDACs in response to the low temperature (4°C). Plants grown at 25°C (x-axis 0) were subjected to cold acclimation (4°C) for 5 days (x-axis 1–5) and then returned to warm temperature (25°C) for 1 day (x-axis 6). Transcript levels were determined by quantitative real-time PCR, and beta actin was used as an internal control. The data are the mean ± standard error for triplicate quantitative PCR reactions for each time point from three independent experiments.

Histone acetylation is a reversible modification that is regulated by two opposing groups of enzymes: HATs and HDACs [23]. Therefore, reduced histone acetylation should be associated with the alteration in HDAC expression. We decided to measure and quantify HDAC messenger RNA levels in control and cold-treated seedlings by real-time quantitative PCR after reverse transcription of RNA. Fifteen HDACs have been indentified in maize. They are encoded by ten maize *RPD3*/*HDA1* genes, one *SIR2* gene and four *HD2*-like genes [24], [25]. *RPD3*/*HDA1* genes and *HD2*-like genes are sensitive to the HDAC inhibitor TSA. *HDA101*, *HDA102* and *HDA108* belong to the *RPD3*/*HDA1* families, and *HDA103* and *HDA106* belong to the *HD2*-like gene family [24]. *RPD3*-type HDAC expression is required for plant development [10], and *HD2* genes were found to be associated with plant resistance to biotic and abiotic stress in barley [24]. Samples were taken from maize seedlings grown on MS medium and treated with cold (4°C) at the indicated time points, and maize seedlings grown under normal conditions (25°C) were used as a control. After cold treatment, plants were again transferred to normal conditions (25°C) and sampled at the indicated time points. We found that HDAC expression levels were rapidly increased immediately after seedlings were transferred to cold stress conditions (Figure 3A–D). These data indicated that cold stress enhanced HDAC expression and further led to global deacetylation of H3 and H4 histones.

### The HDAC inhibitor selectively interferes with cold-induced gene transcription

**Figure 4:**
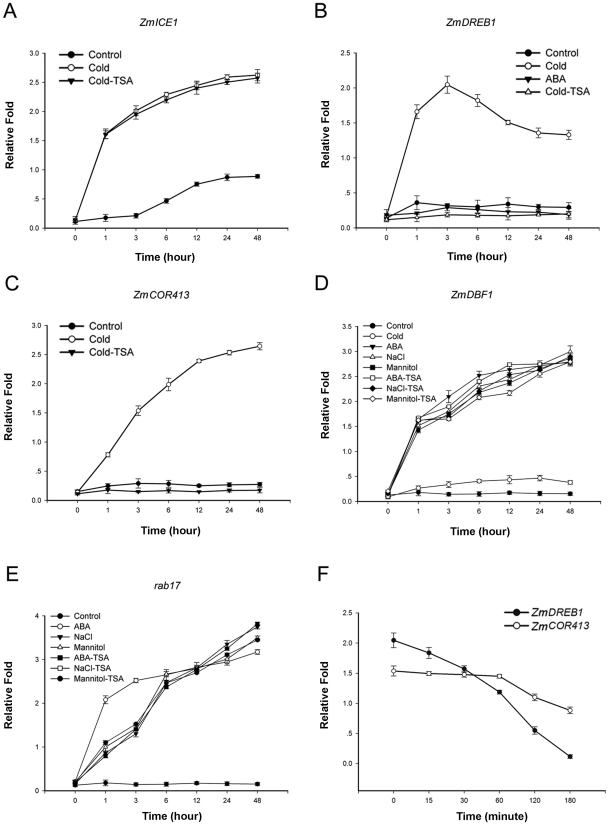
HDACs selectively regulate *ZmDREB1* gene expression. A–C. Transcript levels of stress responsive genes after different treatments were measured by real-time PCR. Compared with the cold-responsive genes *ZmDREB1* and *ZmCOR413*, the expression of *ZmICE1* was not significantly affected by TSA treatment. D–E. *ZmDBF1* and *rab17* were highly induced by ABA, NaCl and mannitol treatments, but not by cold stress. These genes did not demonstrate significant changes in expression after TSA treatment. F. The time course profile of *ZmDREB1* and *ZmCOR413* expression under TSA treatment. TSA treatment rapidly represses *ZmDREB1* and slowly represses *ZmCOR413*. Samples were taken from 7-day-old seedlings grown under 25°C (x-axis 0), and from plants treated with different reagents or stresses at the indicated time points. The data are the mean ± standard error for triplicate quantitative PCR reactions for each time point from three independent experiments.

The above results demonstrated that HDAC expression levels are strongly enhanced by cold stress conditions, implicating HDACs in the cold stress response in plants. Many genes, such as *ICE1*, *DREB1* and *COR413*, are known to rapidly accumulate in plants during cold acclimation [26], [27]. Next, we decided to examine the presence of a relationship between HDACs and these cold-responsive genes at the epigenetic level. We first examined the expression levels of the *ZmICE1* and *ZmDREB1* transcription factor genes. As shown in Figure 4A, rapid up-regulation of the *ZmICE1* gene was observed after cold application, whereas basal levels of *ZmICE1* transcripts could be detected in all control plants. *ZmDREB1* was also highly induced by cold stress; however, it was not induced by ABA application (Figure 4B). Figure 4C shows that the expression of the ZmDREB1 target gene, *ZmCOR413*, was also strongly induced by cold treatment. TSA is an inhibitor of HDACs, and it has been widely used to characterize the function of HDACs in yeasts, mammalian cells and plants [18], [28], [29]. Interestingly, the cold-induced expression of *ZmDREB1* was inhibited after TSA treatment (Figure 4B). However, expression of *ZmICE1* was barely affected by TSA treatment (Figure 4A). As expected, *ZmCOR413* was completely inhibited by TSA (Figure 4C). Substantial evidence shows that cold, drought and high salt stress signals and ABA have some overlapping elements in the signaling network [21]. Drought and salt increase ABA levels and affect gene expression through an ABA-dependent signaling pathway. Therefore, we tested whether HDACs affect ABA-responsive gene expression. *ZmDBF1* is induced by ABA, high salt and drought stress but not by cold, and the *rab17* gene is regulated by *ZmDBF1*. Seedlings were planted on MS medium containing various concentrations of ABA, NaCl and mannitol. As shown in Figure 4D and E, *ZmDBF1* and *rab17* were strongly induced by mannitol, NaCl and ABA treatment. However, cold treatment had a very weak or even no effect on induction of *ZmDBF1*, consistent with the previous results [30]. However, ABA-induced expression of *ZmDBF1* and *rab17* was not affected by TSA treatment (Figure 4D and E). Therefore, these findings indicate that HDACs play an important role in cold-responsive gene expression independently of the ABA pathway, and it positively regulates the expression of these genes.

### HDACs indirectly regulate the expression of the *ZmCOR413* gene

DREB1 binds the CRT/DRE element in the promoter region of the COR gene to activate gene expression [5], [6], and the above results indicated that both *ZmDREB1* and *ZmCOR413* are positively regulated by HDACs. Therefore, we further address whether the decreased expression of *ZmDREB1* and *ZmCOR413* is due to direct TSA-mediated suppression. The TSA-treated time course profiles show that the expression of *ZmDREB1* is repressed within 15 min and down-regulated to an average of 1-fold at 60 min. However, the expression of *ZmCOR413* is not significantly changed during the 60-min time course. These results suggest that the expression of *ZmDREB1* is directly regulated by HDACs, whereas the effect of HDACs on *ZmCOR413* is secondary (Figure 4F).

### TSA-treated plants are hypersensitive to freezing

**Figure 5:**
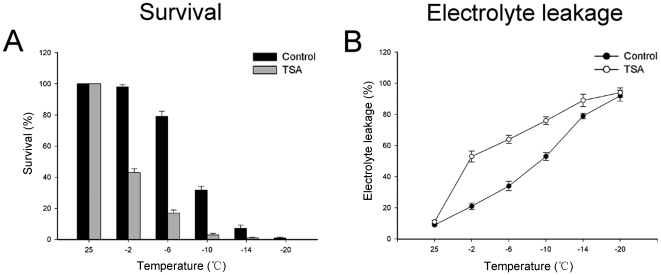
TSA-treated plants are more sensitive to freezing. A. Plants were freeze-treated at the indicated temperatures for 15 minutes and then the survival of plants was quantified. B. Leakage of electrolytes from normal and TSA-treated plants was estimated by determining relative electro-conductivity of the bathing solution of the leaf tissue. Bars represent standard errors.

Because TSA blocks cold-responsive gene transcription, we examined whether the TSA has an effect on the freezing tolerance of plants. Therefore, an electrolyte leakage test was performed. As shown in Figure 5, incubation in 0.5 µM TSA for 24 h decreased cold hardiness in maize seedlings. Normal plants had an LT_50_ value of approximately −9.52°C, whereas TSA-treated plants had an LT_50_ value of approximately −0.98°C. Ion leakage at temperature −20°C represents approximately 100% leakage.

### Cold and TSA give rise to the different histone acetylation statuses across the *ZmDREB1* promoter region

**Figure 6:**
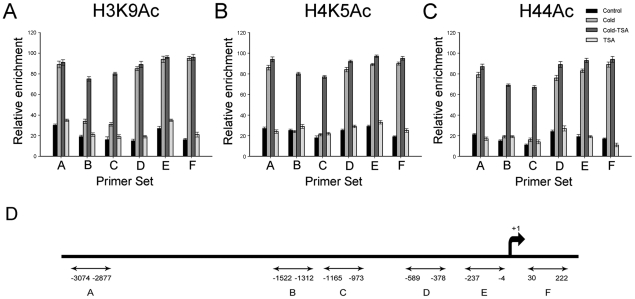
Comparison of H3K9Ac, H4K5Ac and H44Ac levels in the *ZmDREB1* gene region. A–C. Cold stress caused hyperacetylation in the ICE1 binding region (Set A, D and E) and the first exon (Set F). Two other promoter regions (Set B and C) were still hypoacetylated during cold acclimation. When cold-acclimated plants were treated with TSA, all regions were hyperacetylated, and the transcription of *ZmDREB1* was repressed. D. Schematic representation of the *ZmDREB1* gene from −3074 to +222 bp. Primer sets A, D and E contain a MYC recognition element that can be specifically combined with ICE1. Bars represent standard errors.

**Figure 7:**
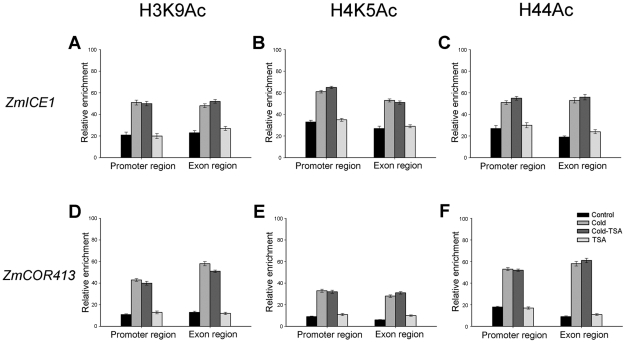
Comparison of H3K9Ac, H4K5Ac and H44Ac levels in the *ZmICE1* and *ZmCOR413* gene regions. Cold stress caused hyperacetylation in the *ZmICE1* and *ZmCOR413* promoter regions and the first exon. Additionally, these two regions were still hyperacetylated after cold-acclimated plants were treated with TSA. No significant changes in histone acetylation were detected in TSA-treated normal plants. Bars represent standard errors.

HATs/HDACs are always recruited to target genes and they cause histone acetylation changes. Thus, we investigated the acetylation state of histones H3 at Lys 9, H4 at Lys 5 and H4 at Lys 5, 8, 12 and 16 across the *ZmDREB1* promoter region by ChIP. Primers encompassing the *ZmDREB1* gene were designed to detect dynamic changes in histone acetylation across the transcriptional regulatory sequences (Table 1). Compared with the histone acetylation levels in control plants, hyperacetylation in cold-acclimated plants occurred in the ICE1 binding region (Sets A, D and E) and a region of the first exon of the gene (Set F) (Figure 6). However, regions adjacent to the ICE1 binding site (Set B and C) remain hypoacetylated after cold treatment (Figure 6). Interestingly, if cold-acclimated plants were treated with TSA, all of these examined regions (Sets A–F) were hyperacetylated (Figure 6). Under normal conditions, the control region of the *ZmDREB1* gene did not undergo significant changes in acetylation levels after TSA treatment (Figure 6). Additionally, histone acetylation of the *ZmICE1* and *ZmCOR413* promoter regions had no significant changes after TSA treatment (Figure 7), which is in agreement with the previous results demonstrating that TSA has no direct effects on these two genes.

**Table 1:** Primers used for quantitative real-time PCR, CHART-PCR and ChIP.

RT-PCR Primer	Sequence (5′-3′)	Efficiency	CHART and ChIP Primer	Sequence (5′-3′)	Efficiency
*HDAC101*	TACTACTCCCTGAATGTCCC	102%	*ZmDREB1A Set A*	GCGAGGCGAGGCAGCTCGGAGTCGG	101%
	TTGAAGCAGCCCAACCTA			GGCGGGCAGGCTGTGCTGGGAGGCT	
*HDAC102*	AACTTTAGATGCTGCTCG	99%	*ZmDREB1A Set B*	TGTCACGGTGGGTGGGTAGGGTGTG	99%
	AAACTCACAGTCATTACCCT			TGAAAGGCCCAATCATATGTGAAAA	
*HDAC103*	GGCAAGACCATTGTAAAC	97%	*ZmDREB1A Set C*	TTCAAACTACGCACATCCATAATTT	102%
	CTATTGGACCTGCGACTC			CAAAACTTAGAACTTGGCTGACCTC	
*HDAC106*	AAAGCGAGTGAGAATGCC	104%	*ZmDREB1A Set D*	ACAAGTTAATGAGCACACCATCACT	97%
	AACAGAGGAACAGGGGAC			AAATTGTAATCTGCATTCGGCAAAC	
*HDAC108*	CGCTTCCACTCCGACGAC	96%	*ZmDREB1A Set E*	ACAGTACAAGGGGCCGCCTAGCAAC	99%
	AGGATGGCGAGGACGATG			GATAGGGTAGCTACCTCTTTGCACT	
*HDAC110*	CCAAGAAAGTCCTCATAGTT	98%	*ZmDREB1A Set F*	TGCTCTGCCACCACCACCTCGTCGT	96%
	TCCAGTTCCAGGGTAAAA			AGCACAGCCTTCCTCCTCCTCCTCC	
*ZmICE1*	CAACCCATCAACACCGAC	102%	*ZmICE1 promoter*	TTTTGCTCTTCAGGCACCTT	99%
	GCAAAGCCATTGAAGCAG			TGGGAAAGAAAACTCGGATG	
*ZmDREB1*	CCAGCGGTAGTTGTTGAC	99%	*ZmICE1 exon*	ACTCGAACAAACCAAATGCC	103%
	TGTTCCCGTTACATTCGT			AAAGATTCGGCTGACAATGG	
*ZmCOR413*	AATGTCCATCTCTATGCGGC	98%	*ZmCOR413 promoter*	ACTCGAACAAACCAAATGCC	101%
	GTACGGCACCACCTTGAGTT			AAACGGATACGCAATAACGC	
*ZmActin*	GATGATGCGCCAAGAGCTG	101%	*ZmCOR413 exon*	ACTCGAACGAACCAAGTGCT	96%
	GCCTCATCACCTACGTAGGCAT			GAAAGCTCAAACCAAGACCG	

### TSA treatment increases the *ZmDREB1* promoter chromatin accessibility to micrococcal nuclease

**Figure 8:**
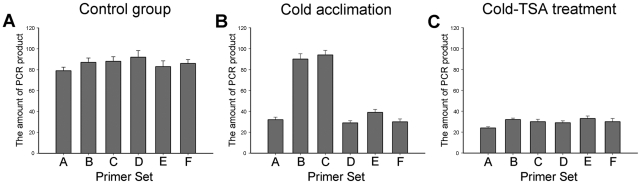
HDACs induced chromatin accessibility at the promoter region of the *ZmDREB1* gene. MNase accessibility by CHART-PCR was performed on nuclei that were extracted from 7-day-old seedlings grown at 25°C (A) and further treated with cold (B) or cold-TSA (C) for 12 h. Primer sets A–F are the same as those used in the ChIP assay. The Ct values generated were converted to PCR product amounts using the standard curve. The y-axis indicates the amount of PCR products and the x-axis indicates the different DNA region. The data shown are the mean and standard errors of triplicate PCRs resulting from three independent experiments.

Histone acetylation/deacetylation usually alters chromatin conformation to regulate gene expression. Therefore, we next examined the effect of TSA on local region chromatin accessibility to micrococcal nuclease (MNase) during cold acclimation. The primer sets were the same as those used in the ChIP assays (Table 1). Decondensed chromatin regions are more accessible to MNase cleavage, and thus MNase can be used to monitor the conformational change of chromatin across a specific region [31]. Increased cleavage occurring across the chromatin region resulted in decreased amplification. Thus, if the treatment of the seedlings induces changes in the chromatin accessibility of MNase, more or fewer PCR products will be generated due to increased or decreased digestion with MNase. As shown in Figure 8A, in untreated seedlings, all measured regions were inaccessible to MNase. In plants cold acclimated for 12 h, regions A, D, E and F were accessible to MNase (Figure 8B), but the other two regions (Sets B and C) remained inaccessible to MNase. In seedlings treated by cold-TSA for 12 h, fewer PCR products were gained due to MNase digestion of all of the regions (Sets A–F) (Figure 8C). Thus, chromatin decondensation may be occurring across the *ZmDREB1* promoter region (B and C) during cold acclimation upon TSA treatment.

### The methylation pattern of the *ZmDREB1* gene 5′ region in TSA and/or cold-treated seedlings

**Figure 9:**
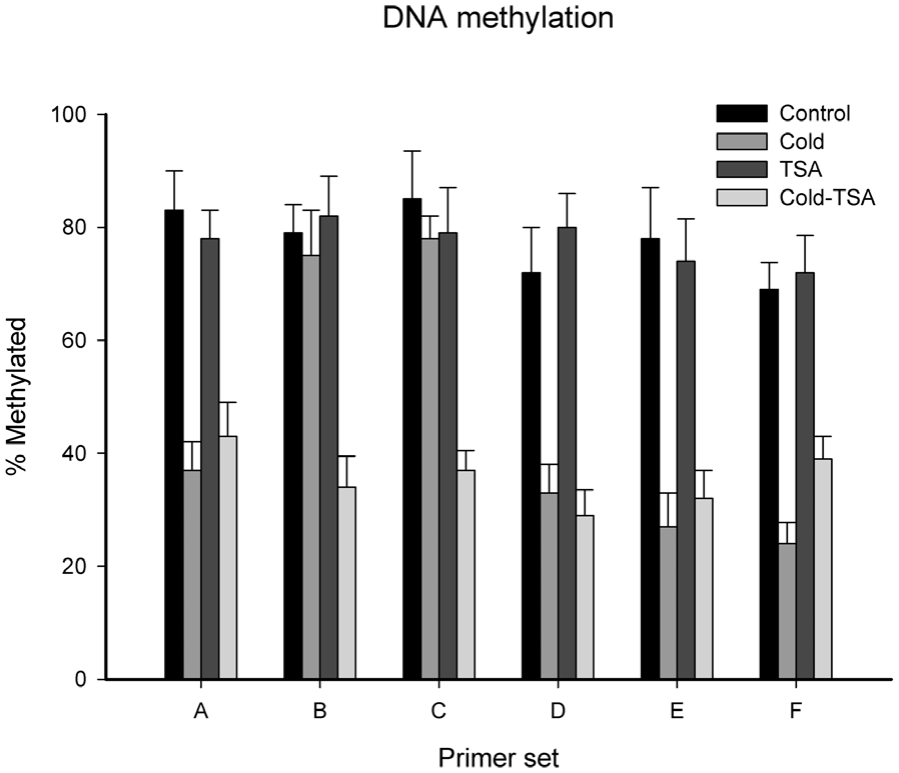
Comparison of the methylation status of CpG motifs in *ZmDREB1* gene region during cold acclimation. Cold stress caused DNA hypomethylation in the ICE1 binding region (Set A, D and E) and the first exon (Set F). Two other promoter regions (Set B and C) were still hypermethylated during cold acclimation. TSA treatment did not significantly affect the DNA methylation status in normal seedlings. However, in cold-TSA treated seedlings, all examined regions (Set A–F) were hypomethylated. Bars represent standard errors.

We further investigated the DNA methylation state of the *ZmDREB1* gene by sodium bisulfite methylation sequencing. Our results indicate that the region of the *ZmDREB1* gene crossing primer sets A through F contains 59 CpG and 44 CpNpG motifs. The mean value of the methylated percentages is shown in Figure 9. Compared with the DNA methylation state in control seedlings, cold stress caused DNA hypomethylation in the ICE1 binding region (Set A, D and E) and the first exon (Set F). Two other promoter regions (Set B and C) were still hypermethylated during cold acclimation. However, in cold-TSA treated seedlings, all examined regions (Set A–F) were hypomethylated (Figure 9). The DNA methylation state was not significantly changed in TSA-treated plants.

## Discussion

We have shown that in maize seedlings, HDACs are highly inducible upon cold treatment (4°C). The increased HDAC expression results in the global deacetylation of histones H3 and H4. Moreover, a histone deacetylase inhibitor selectively suppresses the induction of the cold-responsive transcription factor gene *ZmDREB1* through altering the epigenetic modifications in defined sites of the promoter region. Furthermore, TSA treatment blocks *ZmCOR413*, which is a downstream target, but it does not modulate the expression of several ABA-induced genes. These findings identify HDACs as a signaling component that lies between the *ZmICE1* and *ZmDREB1* transcription factors in the *ICE/DREB1* cold signaling cascade, and it positively regulates cold signal transduction at the chromatin level independently of an ABA signaling pathway. This indicates that this activation function of HDACs is gene and site selective.

### HDACs as a positive signaling component selectively mediated cold-responsive gene expression

A cold-signaling network has been proposed where the cold signal first induces the expression of the *ICE* gene that activates the expression of downstream transcription factor gene *CBF/DREB*. This, in turn, regulates the transcription of downstream target genes [32], [33]. Our results in Figure 4 demonstrate that HDACs might lie between the ZmICE1 transcription factor and the expression of *ZmDREB1*. TSA treatment causes rapid down-regulation of *ZmDREB1*, suggesting that the HDAC functions as a direct transcriptional activator regulating the *ZmDREB1* gene. However, these results cannot exclude the possibility that HDACs may also directly regulate *ZmCOR413* gene expression. Furthermore, treatment with the HDAC inhibitor TSA indicates that the *ZmCOR413* gene is not a direct target of HDACs, instead HDACs appear to directly activate the *ZmDREB1* gene, which then mediates activation of *ZmCOR413*. Therefore, HDACs modulate cold-responsive gene expression as a positive signaling component that directly regulates *ZmDREB1*. Additionally, up-regulation of upstream *ZmICE1* in response to cold stress is less affected by TSA treatment, and HDACs do not regulate ABA-induced gene expression. Together, this indicates that modulation of gene expression by HDACs is gene selective. Similarly, suberoylanilide hydroxamic acid induces p21^WAF1^ expression by selective acetylation of histones associated with p21^WAF1^ [34].

### TSA represses *ZmDREB1* gene expression through altering chromatin modification

HDACs are classically thought to down-regulate or suppress gene expression. Therefore, HDAC inhibitors can up-regulate genes. For example, Arabidopsis AtHD2A, AtHD2B and AtHD2C were shown to repress transcription [35] when targeted to the promoter of genes, and Arabidopsis HOS15 repressed *RD29A* expression through *RD29A* promoter-associated histone deacetylation [8]. Similarly, TSA induces up-regulation of 45 genes during Arabidopsis seed germination [36]. However, we show here that *ZmDREB1* and *ZmCOR413* were rapidly down-regulated when HDAC activity is perturbed by TSA, indicating that HDACs may also activate the transcription of cold-responsive genes in maize. This finding is supported by the similar observation that overexpression of *HDA19* in Arabidopsis results in up-regulation of ERF1 [10]. The study in yeast indicates that HDACs are essential for efficient induction of some genes. For example, RPD3 has been shown to activate telomeric genes [18]. In animal culture cells, HDAC inhibitors decrease the expression of some cytokine genes [20].

Histone acetylation/deacetylation at the promoter regions of some genes is usually involved in the alteration of the local chromatin conformation that regulates gene expression, and our findings agree with this idea. ChIP and CHART-PCR data indicate that the acetylation level and the chromatin status of the ICE1 binding regions remain unchanged in cold-acclimated plants after TSA treatment, implying that TSA has no effect on these regions. Additionally, ICE1 may remain bound to these regions in the promoter. However, in cold-acclimated plants, chromatin decondensation was observed at other two regions in the promoter after TSA treatment, and this selective chromatin conformational change appears to facilitate the binding of certain factors. The results suggest that TSA inhibits the expression of *ZmDREB1* through changing chromatin modification in defined sites that regulate expression of this gene. Therefore, we speculate that HDACs likely regulate *ZmDREB1* gene expression by preventing binding of certain factors through maintaining a deacetylated state in these regulatory regions. Identification of the unknown repressor and related cis-acting elements of the *DREB1* gene will confirm this speculation. A similar situation has been described for activation of telomeric genes in yeast through deacetylation of histones by Rpd3p, which then prevented binding of the SIR repressor proteins [18]. HDACs may also activate transcription via chromatin remodeling, enabling transcription factor recruitment. For example, chromatin remodeling by HDACs activates proinflammatory gene expression through recruitment of transcription factors [20]. Furthermore, our recent study indicates that a mutual reinforcing action between histone acetylation, histone methylation and DNA methylation occurs during maize mitosis [37]. DNA methylation has been reported to control methylation of H3K9 and heterochromatin assembly in *Arabidopsis* [38], [39]. In *Neurospora*, TSA could cause selective loss of DNA methylation [40]. Some reports suggested that histone H4 hyperacetylation could affect DNA methylation levels [41], [42]. It was also reported that cold stress induces DNA demethylation in maize [43]. Thus, multiple modifications of histones and DNA may co-function in various combinations in response to environmental stresses.

Our results indicate that HDACs cause global histone deacetylation in response to cold stress and regulate the expression of *ZmDREB1* and *ZmCOR413*. TSA suppresses cell cycle progression by causing hyperacetylation of histones in tobacco protoplasts, [44] and it can delay seed germination through a global deacetylation event [36]. Studies in yeast show that the overall acetylation/deacetylation processes may function to control basal transcription [45]. Global acetylation and deacetylation also allow for rapid restoration of acetylation levels when the recruited HAT or HDAC is removed [45], [46]. Therefore, we further extend these findings to show that cold induces a hypoacetylated state through the genome, and global acetylation/deacetylation may be involved in rapid reversal of transcription levels after the removal of cold stress.

## Materials and Methods

### Plant materials and treatments

Maize seeds (*Zea mays* L. inbred line Huangzao 4) were sterilized and grown in 1/5 diluted Murashige and Skoog medium (MS) under continuous light (120 µmolm^−2^s^−1^) for 7 days at 25°C and 70% relative humidity in a growth cabinet [43]. To investigate the effects of cold stress, ABA, NaCl, mannitol and trichostatin A (TSA), nine experimental groups were formed. Each experimental group contained 30 seedlings. Seedlings in the control group remained in 1/5 diluted MS medium and were kept in a 25°C incubator for the entire length of the experiment. For cold treatment, seedlings in the “cold” group were transferred to a 4°C incubator and grown in 1/5 diluted MS medium for 4 days. After cold stress, the seedlings were grown for another 4 days in the 25°C incubator for recovery. For the ABA, salt and drought treatments, seedlings belonging to the “ABA”, “NaCl” and “mannitol” groups were transferred to 1/5 diluted MS medium containing 100 µM ABA, 300 mM NaCl or 250 mM mannitol, respectively and then grown in a 25°C incubator for 48 hours. For TSA treatment, seedlings in the “TSA” and “cold-TSA” groups were incubated in 1/5 diluted MS medium containing 0.5 µM TSA at 25°C and 4°C, respectively. For “ABA-TSA”, “NaCl-TSA” and “mannitol-TSA” treatments, 7-day-old seedlings were transferred to 1/5 diluted MS medium containing 0.5 µM TSA combined with 100 µM ABA, 300 mM NaCl or 250 mM mannitol, respectively and cultivated in a 25°C incubator for 48 hours. Light and humidity conditions were kept constant throughout the experimental periods.

### Antibodies

The following antibodies were used for immunostaining and ChIP. From upstate (Lake Placid, NY, USA): H3K9ac (catalog number 07-352), H4K5ac (catalog number 06-759), H44ac (catalog number 06-866), Anti-Histone H3 (catalog number 06-755), and fluorescein-conjugated goat anti-rabbit IgG (catalog number 16-237). From Sigma (St. Louis, MO, USA): AP-conjugated goat anti-rabbit IgG (catalog number A4187).

### Western blotting assays

Proteins were extracted by grinding samples in liquid nitrogen and resuspended in extraction buffer (100 mM Tris-HCl pH 7.4, 50 mM NaCl, 5 mM EDTA and 1 mM PMSF). Western blot detection was carried out as previously described [47]. Actin and histone H3 were used as equal loading controls. Western blots were repeated three times for each sample from three independent experiments.

### Flow cytometric assays

Nuclei were prepared according to the method described by Li et al. [48]. The cell-cycle profile was determined with a FACSCalibur flow cytometer (Becton Dickinson, San Jose, CA, USA) equipped with an argon-ion laser, using the 488 nm laser line for excitation. CellQuest software running on an Apple Macintosh computer connected to the flow cytometer was used for data acquisition.

### Immunostaining

Immunostaining of nuclei was performed according to the method described by Yang et al. [37]. Nuclei were spread on a slide, incubated with the primary antibody at 4°C overnight, followed by 2 h incubation at 37°C with the secondary antibody. In control experiments, slides were incubated with only the secondary antibody. All slides were counterstained with DAPI (Sigma, St. Louis, MO, USA), mounted with Vectashield (Vector labs, Burlingame, CA, USA) and examined with an Olympus BX-60 fluorescence microscope with filter blocks for DAPI and fluorescein. Images captured with a CCD monochrome camera Sensys 1401E were pseudo-colored and merged using the software MetaMorph® 4.6.3 (Universal Imaging Corp., Downingtown, PA, USA). Microscope settings and camera detector exposure times were kept constant for each respective channel (fluorescein or DAPI) but were optimized for individual experiments. More than 500 nuclei were analyzed for each treatment group. For both control and treated groups, three independent immunostaining experiments were performed with each antibody. Image J and MetaMorph measured the mean gray values of the signal intensity, and data were analyzed by SPSS10.0.

### Reverse transcription and real-time PCR

Total RNA was isolated using the RNAprep pure Plant Kit (Qiagen, Mannheim, Germany) according to the manufacturer's instructions. To remove residual DNA contamination, 1 µg of total RNA was treated with 50 units of DNase I (Fermentas, Burlington, ON, Canada) at 37°C for 30 min. The purified RNA was reverse-transcribed into cDNA with the RevertAid First Strand cDNA Synthesis Kit (Fermentas, Burlington, ON, Canada), as recommended by the manufacturer.

Quantitative real-time PCR was performed using SYBR® Green Real-time PCR Master Mix (Toyobo, Tokyo Japan) on the Rotor-Gene 2000 Real-Time Cycler (Corbett Research, Mortlake, NSW, Australia). For all real-time PCR assays, standard curves were generated for each primer set to determine their efficiency, and melting curves were generated to detect non-specific amplification products and primer-dimers. The PCR product was also separated on a 2% agarose gel to confirm its size (<300 bp), and it was also sequenced to verify its identity. In the preliminary experiment, we tested the expression of several reference genes, such as actin (GenBank accession number: J01238), 18S (GenBank accession number: EU975801.1) and GAPDH (GenBank accession number: X07156.1), in the control and treatment seedlings, and we observed that actin transcription (Ct values: 28.323±0.074) was the most stable because it was not significantly regulated or influenced by the experimental procedure. Therefore, the actin gene was selected as a reference gene in this study. Triplicate PCR reactions for each of the three independently-purified RNA samples were carried out. Template-free and SYBR Green mix-free samples were amplified for each gene as negative controls. The standard amplification conditions were: 95°C for 30 sec, followed by 45 amplification cycles at 95°C for 10 sec, 58–60°C for 10 sec, and 72°C for 15 sec. Fluorescence data were acquired at the 72°C step and during the melting-curve program. The threshold cycle numbers (Ct) for each PCR product was determined, and the relative expression levels for all genes were obtained using 2^−ΔΔCt^ calculations by Rotor Gene software version 6.0.19 (Corbett Research, Mortlake, NSW, Australia). Quantitative PCR primers were designed using the Primer Premier 5 software. These primer sequences and the reaction efficiencies (ranging from 96% to 104%) are detailed in Table 1.

### Chromatin immunoprecipitation (ChIP) assay

The ChIP assay was carried out with H3K9Ac, H4K5Ac and H44Ac antibodies following the procedure described by Haring et al. [49]. During the ChIP assay, a negative control was performed using rabbit serum for mock immunoprecipitation. After ChIP, DNA was extracted with a standard procedure (phenol/chloroform/isoamyl alcohol) (25∶24∶1). Precipitated genomic DNA was subjected to real-time PCR with primer sets A–F (Table 1) encompassing the promoter region (A–E) and the first exon (F) of the *DREB1* gene. Quantitative Real-time PCR was performed according to the above-mentioned procedure.

### Chromatin accessibility measured by CHART-PCR

To analyze the chromatin conformational change, a chromatin accessibility by real-time PCR (CHART-PCR) assay was carried out essentially according to the method described by Rao et al. [50]. Seedlings were cold or cold-TSA treated, and nuclei were prepared as described by Lo et al. [51]. Nuclei were then digested for 5 min at 37°C using 5 U micrococcal nuclease (MNase). Subsequently, DNA was prepared using a Plant genomic DNA kit (Qiagen, Mannheim, Germany) and quantified using the Gene Quant calculator (Amersham Pharmacia Biotec, Piscataway, NJ, USA). One hundred nanograms of genomic DNA from cold or cold-TSA-treated samples was used for SYBR Green real-time PCR analysis with the same primer sets that were used for the ChIP assay (Table 1). MNase accessibility is thought to be inversely proportional to the amount of amplified product.

### Assessment of freezing tolerance

Normal and TSA group (0.5 µM TSA treated for 24 h) plants were used for cold acclimation tests, and electrolyte leakage analysis was performed following the method described by Ristic and Ashworth [52]. For survival analysis, 15 plants from each group were freeze-treated at each indicated temperatures (−2, −6, −10, −14 and −20°C) for 15 minutes, and then the mean relative survival of 15 seedlings was determined. For the electrolyte leakage test, 25 fully expanded leaves were taken from 15 plants of each group and placed in glass-stoppered test tubes (1 leaf per tube containing 4 ml deionized water). After a 30 min equilibration period at 4°C, the bath temperature was reduced at 2°C/h rate to −20°C (Masterline Model 2095, Forma Scientific, Marietta, OH, USA). Five tubes from each group were withdrawn at each indicated temperature (−2, −6, −10, −14 and −20°C) and placed on ice overnight. Subsequently, the electro-conductivity of the bathing solution was measured using a conductance meter (YSI model 35, Yellow spring, Ohio, USA). A value set for 100% leakage was obtained by boiling the sample for 1 h. The electro-conductivity before boiling was calculated as a percentage compared to the levels after boiling to give relative electro-conductivity. Then, the average relative electro-conductivity of 5 leaves was calculated. The temperature value for 50% electrolyte leakage, which was defined as the LT_50_, was determined by a plot of freezing temperature versus relative electro-conductivity.

### Bisulfite sequencing

The methylation status of CpG and CpNpG motifs in the *ZmDREB1* promoter region was determined using bisulfite sequencing. Approximately 2 µg of genomic DNA was denatured in 0.3 M NaOH (30 min, 20°C), neutralized with ammonium acetate, and ethanol precipitated. Nonmethylated cytosines were deaminated in 1.5 ml of 4 M NaHSO_3_ and 500 µM hydroquinone at 55°C for 16 h. Subsequently, DNA was purified by gel filtration, incubated in 0.3 M NaOH (10 min, 37°C), and ethanol precipitated. Purified DNA was resuspended in 50 µl of ddH_2_O. Finally, DNA was analyzed using primer sets spanning a region from −3074 to +222 bp of the *ZmDREB1* gene. PCR products were purified using a gel extraction kit (Qiagen, Mannheim, Germany) and were directly sequenced using an automated DNA sequencer. Each sample was sequenced 3 times to determine site-specific methylation changes in the amplified regions. To ensure that only bisulfite-reacted DNA was amplified and to avoid biased amplification of methylated strands, primers spanning these regions were designed using methprimer software (Table 2).

**Table 2:** Primers used for bisulfite sequencing.

Bisulfite sequencing Primer	Sequence (5′-3′)
*ZmDREB1A Set A*	TTTTAATTATATGTTTATGTTTTGTAGGT
	ACCCTACCCTCACCCTATCATATAT
*ZmDREB1A Set B*	GGGTATATGAGGTTTTTTTGTTA
	ATATAAACTCTTTAATCTCACCTTACCTC
*ZmDREB1A Set C*	TGTTAATAGTTTTTTTTATGGGTGGAG
	CCACAAACAACACAAACATAATCTTATAT
*ZmDREB1A Set D*	CACACCATCACTACTCACTA
	TTCGGCAAACTATACAACAC
*ZmDREB1A Set E*	AAAATTGTGGGATAGTATAAGGGGT
	CAAAAAAATCAAATAATCAAAAAACTAATT
*ZmDREB1A Set F*	TCACAGACTCGTCCTCTT
	ATTACCAGCACAGCCTTC
